# Macroautophagy Proteins Assist Epstein Barr Virus Production and Get Incorporated Into the Virus Particles

**DOI:** 10.1016/j.ebiom.2014.11.007

**Published:** 2014-11-08

**Authors:** Heike Nowag, Bruno Guhl, Kerstin Thriene, Susana Romao, Urs Ziegler, Joern Dengjel, Christian Münz

**Affiliations:** a Viral Immunobiology, Institute of Experimental Immunology, University of Zürich, 8057 Zürich, Switzerland; b Center for Microscopy and Image Analysis, University of Zürich, 8057 Zürich, Switzerland; c Department of Dermatology, Medical Center, University of Freiburg, Hauptstr. 7, 79104 Freiburg, Germany; d Freiburg Institute for Advanced Studies, University of Freiburg, Albertstr. 19, 79104 Freiburg, Germany; e BIOSS Centre for Biological Signalling Studies, University of Freiburg, Schänzlestr. 18, 79104 Freiburg, Germany; f ZBSA Center for Biological Systems Analysis, University of Freiburg, Habsburgerstr. 49, 79104 Freiburg, Germany

**Keywords:** EBV, Epstein Barr virus, Atg, autophagy related gene, BMRF1, BamH1 M fragment rightward reading frame 1, BZLF1, BamH1 Z fragment leftward reading frame 1, BHRF1, BamH1 H fragment rightward reading frame 1, BALF1, BamH1 A fragment leftward reading frame 1, BALF4, BamH1 A fragment leftward reading frame 4, BRLF1, BamH1 R fragment leftward reading frame 1, BNRF1, BamH1 N fragment rightward reading frame 1, EBNA1, Epstein Barr virus nuclear antigen 1, LMP1, latent membrane protein 1, vFLIP, viral FLICE-like inhibitor protein, Atg8/LC3, Atg12, Atg16, BZLF1, Lytic EBV replication, Epithelial cell, B cell

## Abstract

Epstein Barr virus (EBV) persists as a latent herpes virus infection in the majority of the adult human population. The virus can reactivate from this latent infection into lytic replication for virus particle production. Here, we report that autophagic membranes, which engulf cytoplasmic constituents during macroautophagy and transport them to lysosomal degradation, are stabilized by lytic EBV replication in infected epithelial and B cells. Inhibition of autophagic membrane formation compromises infectious particle production and leads to the accumulation of viral DNA in the cytosol. Vice versa, pharmacological stimulation of autophagic membrane formation enhances infectious virus production. Atg8/LC3, an essential macroautophagy protein and substrate anchor on autophagic membranes, was found in virus preparations, suggesting that EBV recruits Atg8/LC3 coupled membranes to its envelope in the cytosol. Our data indicate that EBV subverts macroautophagy and uses autophagic membranes for efficient envelope acquisition during lytic infection.

## Highlights

• Macroautophagic membranes are stabilized during lytic EBV replication.
• Inhibition of macroautophagic membrane formation reduces EBV production.
• Stimulation of macroautophagic membrane formation boosts EBV production.
• Without macroautophagic membranes EBV DNA accumulates in the cytosol.
• Macroautophagic membranes get incorporated into EBV particles.

## 1 Introduction

Epstein Barr virus (EBV), a γ-herpes virus, is a remarkably successful human pathogen with more than 95% of the entire adult human population being infected (Rickinson et al., 2014). 50 years ago, EBV was discovered as the first human tumor virus candidate in tissue samples of Burkitt's lymphoma patients (Epstein et al., 1964). Cell-mediated immune control is thought to keep persistent EBV infection in check and prevent virus-associated malignancies. These malignancies harbor latent EBV proteins, which drive cellular proliferation and survival in order for EBV infected B cells to gain access to the memory B cell pool, the virus' site of persistence (Babcock et al., 1998, Kutok and Wang, 2006). EBV can reactivate from this memory B cell pool into lytic replication, and infectious particle production is initiated upon plasma cell differentiation (Laichalk and Thorley-Lawson, 2005). The resulting virions are similar to those of other members of the herpes virus family. EBV's double stranded DNA is encased by an icosahedral nucleocapsid, sheltered by a protein tegument and ultimately surrounded by a viral envelope decorated with viral glycoproteins (Johannsen et al., 2004). A primary viral envelope is acquired after nucleocapsid assembly via budding through the inner nuclear membrane. This first envelope is shed after fusion with the outer nuclear membrane, thereby releasing the de-enveloped nucleocapsid into the cytosol. This nucleocapsid acquires then its second and final envelope in the cytosol. It is thought that the membrane source of the final herpes virus envelope is perinuclear organelles like the trans-Golgi-network and often requires virus induced organelle reorganization (Henaff et al., 2012). The molecular machinery of macroautophagy can achieve such membrane remodeling, and the second enveloping of EBV is topologically reminiscent of macroautophagy. Indeed, α- and β-herpes virus infections are able to induce macroautophagy (English et al., 2009, McFarlane et al., 2011), and herpes simplex virus (HSV) particles can be found in autophagic vesicles (English et al., 2009). However, no requirement of macroautophagy for the replication of these α- and β-herpes viruses was reported so far.

Macroautophagy is a highly conserved major cytoplasmic degradation pathway of eukaryotic cells, which, in contrast to the ubiquitin–proteasome system, can engulf protein aggregates and organelles in a double-membrane vesicle. This so-called autophagosome fuses with lysosomes in order to degrade its cargo (Mizushima et al., 2011). The molecular machinery required for macroautophagy consists of more than 30 autophagy-related gene (atg) products. Among these, Atg8 – in mammalian macroautophagy studies mainly represented by its LC3B ortholog – gets coupled to the autophagic membrane. Atg8/LC3 mediates membrane fusion events during vesicle formation and recruits cargo via LC3-interacting region (LIR) containing proteins. The ubiquitin-like membrane coupling of Atg8/LC3 to membranes is mediated by the ligation complex of Atg12, Atg5 and Atg16. In this complex the ubiquitin-like molecule Atg12 is covalently attached to Atg5 and assembles with Atg16 to a protein shell cradling the emerging autophagosome (Kaufmann et al., 2014). This complex is frequently targeted to inhibit macroautophagy. All Atgs dissociate from the outer membrane upon autophagosome completion to allow efficient fusion with lysosomes. Various organelles have been implicated as a source of autophagic membranes, including endoplasmic reticulum, Golgi apparatus, outer nuclear membrane, mitochondria and the plasma membrane (Lamb et al., 2013). Therefore, Atgs can restructure cellular membranes and might be involved in cytosolic enveloping of herpes viruses.

Thus, the aim of this study was to investigate the role of macroautophagy proteins during EBV replication. We demonstrate that in the transition from latent to lytic EBV infection autophagic membranes are stabilized. Inhibition of this accumulation compromises infectious particle production and arrests viral DNA export from the cytosol. Furthermore, Atg8/LC3-conjugated membranes get incorporated into mature virions. These data suggest that EBV uses Atg8/LC3-coupled membranes for cytosolic enveloping.

## 2 Results

### 2.1 Autophagic Membranes are Stabilized Upon Lytic EBV Replication

**Fig. 1:**
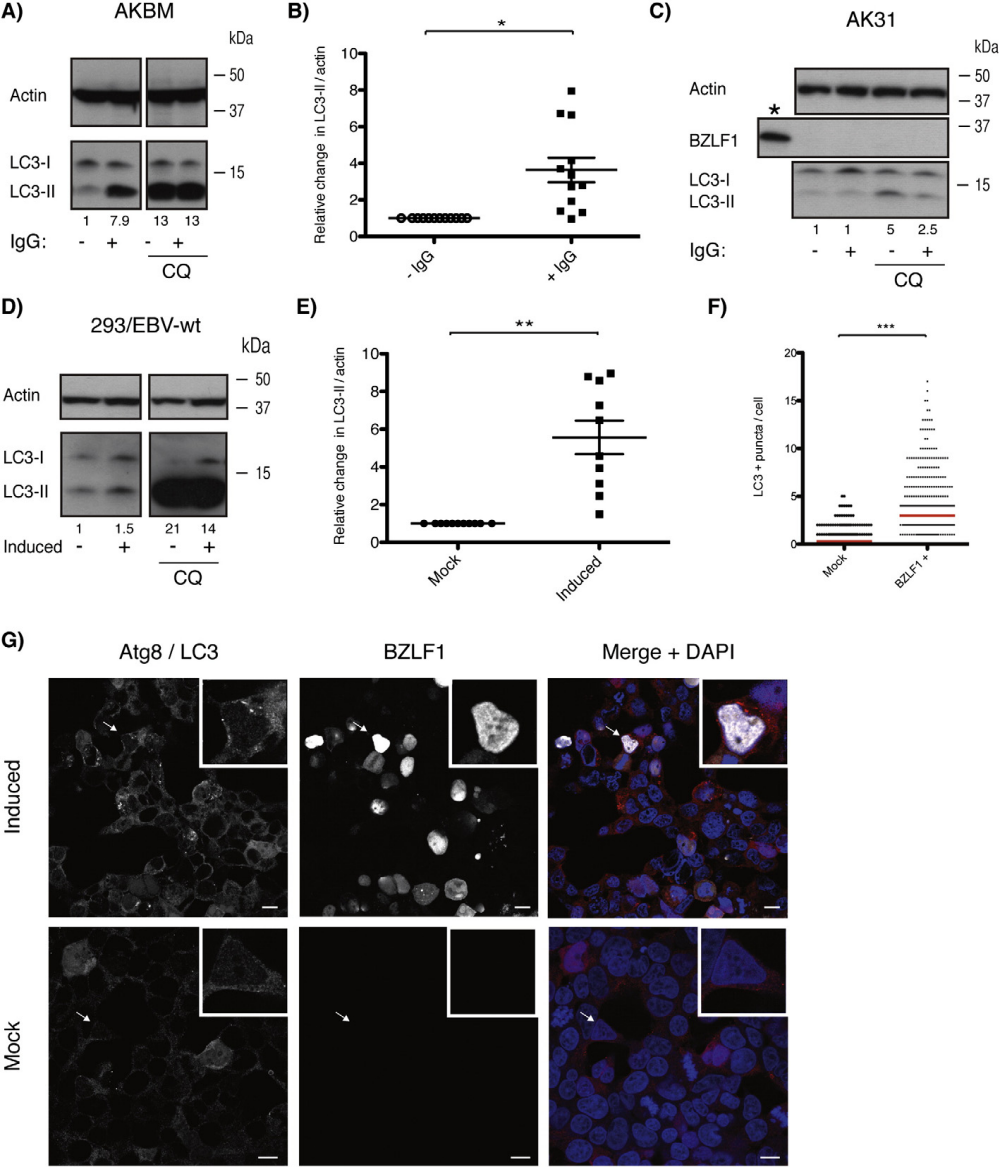
Autophagic membrane accumulation in EBV producing cells. (A) Human AKBM cells were incubated with anti-human IgG F(ab)_2_ fragments for 2 h. Atg8/LC3 and β-actin protein levels were assessed after 20 h by Western blot. In parallel, 50 μM chloroquine (CQ) was supplemented during the final 6 h of incubation. Numbers below blots indicate fold change of LC3-II/β-actin ratio normalized to latent AKBM sample. Blot represents 12 individual experiments. (B) Densitometry quantification of LC3-II bands normalized to their corresponding β-actin levels in latent (− IgG) and lytic (+ IgG) AKBM cells (n = 12). Data are expressed as means ± SD; paired *t* test; *, P < 0.05 on RU values of each blot. (C) Human AK31 cells were incubated with anti-human IgG F(ab)_2_ for 2 h. Atg8/LC3, BZLF1 and β-actin protein levels were examined 20 h post-ligation by Western blot. 50 μM CQ was supplemented in the final 6 h of incubation, in parallel. Asterisk denotes positive BZLF1 protein band of AKBM sample. Numbers below blot indicate fold change of LC3-II/β-actin ratio normalized to non-induced Ak31 sample. Blot represents one of 3 individually performed experiments. (D) 293/EBV-wt cells were analyzed 3 days post-transfection for Atg8/LC3 and β-actin by means of Western blot analysis. In parallel, 50 μM chloroquine (CQ) was supplemented in the final 6 h of incubation. Blot is representing one of 10 individual experiments. (E) Densitometry quantification of LC3-II bands normalized to β-actin in mock and EBV-producing 293/EBV-wt cells (n = 10). Data are expressed as means ± SD; paired *t* test; **, P < 0.01 on RU values of each blot. (F) Quantification of LC3 positive vesicles in BZLF1 expressing cells undergoing lytic EBV production versus mock 293/EBV-wt cells. (n(mock) = 373; n(BZLF1^+^) = 369). Data expressed as median ± SD; *t* test; ***, P < 0.001. Transfection efficacy was at least 50% as assessed by BZLF1 positivity. (G) Immunofluorescence representations of 293/EBV-wt cells 3 days post-transfection. Staining for BZLF1 (white), Atg8/LC3 (red) and DAPI (blue), scale bar 10 μm. (DAPI, 4′,6-diamidino-2-phenylindole).

We used two cellular systems to investigate macroautophagy regulation in the transition from latent to lytic EBV infection. One is based on the Burkitt's lymphoma cell line Akata (Takada et al., 1991), and was transfected with ratCD2–GFP under the early lytic BamH1 M fragment rightward reading frame 1 (BMRF1) promotor (Ressing et al., 2005). These AKBM cells initiate lytic EBV replication upon B cell receptor (BCR) cross-linking, resulting in the expression of GFP and ratCD2 on their surface (Supplementary Fig. 1). In order to study macroautophagy during lytic replication, membrane conjugation of the autophagosome marker Atg8/LC3 (LC3-II) (Klionsky et al., 2012) was monitored 20 h post-ligation of the BCR on AKBM and on control AK31 (EBV-negative and reporter construct transfected Akata cells) (Fig. 1A and C). Latent AKBM cells showed moderate levels of LC3-II, which increase 4-fold upon the induction of lytic EBV replication (Fig. 1B). In contrast to this late up-regulation of macroautophagy (20 h post-induction), LC3-II levels were lower than in the non-induced AKBM control at early time points (6 h post-induction), indicating a blockade or reduction in autophagy. These protein levels were marginally enhanced within 12 to 16 h, but after an incubation of 24 h, the LC3-II protein levels had risen significantly above the steady-state levels (Supplementary Fig. 4A). When narrowing down the time frame of macroautophagy upregulation, at 13 h macroautophagy was still unaffected, whereas the 17-hour time point depicted a change in autophagy and the respective up-regulation was even stronger when analyzing LC3-II protein levels after 21 h (Supplementary Fig. 4B). In contrast, AK31 cells did not show any alterations in endogenous LC3-II levels in response to the ligation of surface IgG (Fig. 1C). Importantly, these cells also did not display any expression of the BamH1 Z fragment leftward reading frame 1 (BZLF1) protein upon cross-linking, verifying that these cells are indeed EBV negative and do not enter lytic EBV replication, which is initiated by BZLF1 (Fig. 1C). In addition, the production of human IL-10 into the supernatant of IgG treated AKBM and AK31 verified successful BCR ligation on both cell types (Supplementary Fig. 2). Up-regulation of LC3-II expression can derive from increased macroautophagy, or from an accumulation of autophagosomes. In order to distinguish these two possibilities we blocked lysosomal degradation of autophagosomes. Chloroquine is a lysosomotropic reagent elevating the pH within lysosomes and therefore inhibits the degradation of autophagosomes. Upon treatment with chloroquine, LC3-II accumulation could be observed in latent and lytic cells to the same levels, indicating that the detected upregulation of LC3-II expression by lytic EBV replication was a result of a partial block of autophagic membrane delivery to lysosomes (Fig. 1A). Moreover, using immunofluorescence approaches, we observed that reactivation to the lytic phase induced autophagosome accumulation in the cytosol of AKBM cells (Supplementary Fig. 3). Therefore, EBV infected B cells stabilize autophagic membranes upon lytic EBV reactivation.

In order to study another EBV infected cell type, we assessed alterations in macroautophagy in the 293/EBV-wt epithelial cell line, which harbors the recombinant EBV genome of the B95-8 strain and can be induced to produce EBV particles via BZLF1 and BALF4 transfection (Delecluse et al., 1998). In line with the results obtained in the AKBM cell line, a 5.5-fold increase in LC3-II protein levels was observed in transfected 293/EBV-wt cells compared to mock transfected cells 3 days post-induction (Fig. 1D and E). In both 293/EBV-wt and AKBM cells lipidated LC3 accumulated after more than 20 h of lytic EBV cycle induction (Supplementary Fig. 4C). Increased macroautophagy could also be observed by the quantification of LC3 puncta formation in the cytoplasm of BZLF1 positively stained, lytic 293/EBV-wt cells (Fig. 1F and G). Additionally, LC3 punctae were already visible within 24 and 48 h post-transfection in 293/EBV-wt cells (Supplementary Fig. 5). Thus, these results suggest that lytic EBV replication leads to the accumulation of autophagic membranes by partially blocking their degradation.

### 2.2 Macroautophagy Modulates the Production of Infectious EBV Particles

**Fig. 2:**
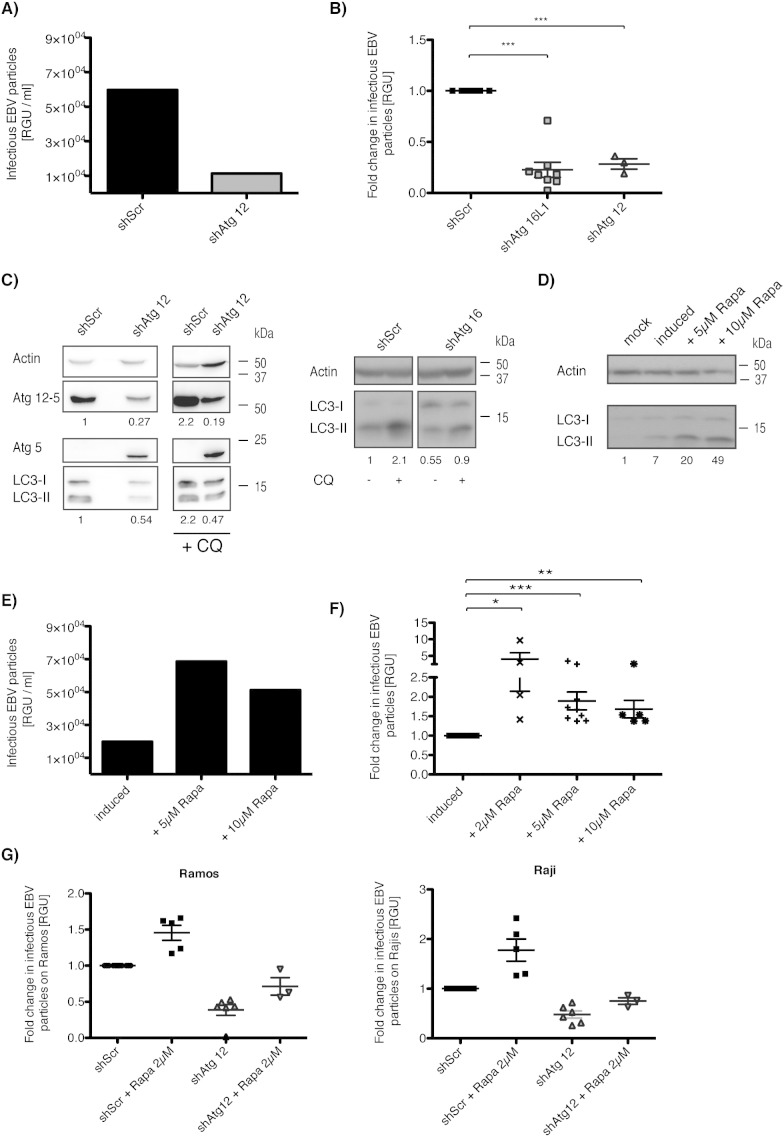
Modulation of macroautophagy in 293/EBV-wt cells influences the production of infectious EBV particles. (A) 293/EBV-wt cells were transduced with lentiviral atg12 specific (shAtg12) or control shRNA (shScr). Infectious EBV production was assessed in scramble versus Atg12 specific shRNA expressing cells by Raji titration and is represented in RGU/ml. The results are representative of three independent experiments. (B) Quantification of RGU/ml in individual experiments. Data is represented in fold change in RGU normalized to control (shScr). Data are expressed as means ± SD; paired *t* test; ***, P < 0.001 on RGU/ml in individual experiments (shAtg16, Atg16 specific shRNA). (C) Atg12 and Atg16 silencing levels for (A and B) were tested by immunoblot, probing for LC3, Atg12–Atg5 complexes and β-actin on day 3 after transfection and quantified by densitometry. In parallel, 50 μM chloroquine (CQ) was supplemented in the final 6 h of incubation. Left: Atg12 silencing efficacy assessed by Atg12–Atg5 complex, Atg5 and LC3-II protein levels. Quantification of Atg12–Atg5 complex normalized to β-actin below Atg12–Atg5 blot and ratio LC3-II/β-actin below LC3-II blot, Scramble set to 1. Right: Atg16 silencing tested by immunoblot for LC3 and actin. Numbers below blot denote the quantification of LC3-II protein levels normalized to β-actin, with scramble set to 1. (D) Representative Western blot for rapamycin treatment of (E). Quantification indicated by numbers below the blot represents the fold increase of LC3-II compared to mock cells after normalization to the corresponding β-actin levels. One representative blot out of three performed is shown (Rapa, rapamycin). (E) 293/EBV-wt cells were incubated with rapamycin (Rapa) 4 h prior to transfection. Raji titration was used to analyze EBV content in supernatants of treated versus untreated lytic 293/EBV-wt cells 3 days post-transfection. Representative Raji titration is shown and represents 5 independently performed assays. (F) Quantification of Raji titrations performed on varying concentrations of rapamycin treated 293/EBV-wt cells. Data is expressed in fold change of rapamycin treated groups compared to lytic, untreated. Data expressed as means ± SD; paired *t* test on RGU numbers; *, P < 0.05, **, P < 0.01 ***, P < 0.001. (G) Quantification of Ramos and Raji titrations with same supernatants applied for each cell type. Data represents at least 3 independently performed assays and is expressed as means ± SD.

In order to investigate the influence of macroautophagy on EBV maturation and viral particle production, we silenced macroautophagy gene expression via lentiviral shRNA constructs, targeting Atg12 and Atg16 in 293/EBV-wt cells. The Atg12–Atg5–Atg16 complex is essential for autophagosome formation by ligating Atg8/LC3 to autophagosomal membranes (Fujita et al., 2008, Hanada et al., 2007). Recombinant B95-8 EBV production by 293/EBV-wt cells could be quantified in subsequent titrations on Raji and Ramos cells, since this virus contains a GFP reporter cassette, which gets rapidly expressed in infected cells and can be quantified as Raji or Ramos green units (RGU). Atg12 and Atg16 silencing reduced the production of EBV particles from 293/EBV-wt cells during lytic EBV infection by 60–80% in the supernatants as assessed by Raji and Ramos titration (Fig. 2A, B and G). Notably, this macroautophagy inhibition did not alter cell viability in transduced cells compared to controls (Supplementary Fig. 6).

Furthermore, in line with these loss-of-function experiments, we could observe the opposite effect for rapamycin-induced macroautophagy during EBV production. Infectious particle production was increased upon pharmacological macroautophagy stimulation (Fig. 2E to G). Although rapamycin treatment enhanced virus production and macroautophagy, concentration higher than 5 μM did not increase virus production further (Fig. 2E and F), and might even overcome the block of autophagosome maturation during lytic EBV infection, resulting in virus degradation. The rapamycin induced enhanced infectious particle production was dependent on autophagic membrane formation, because Atg12 silencing abolished the rapamycin induced increase in infectious EBV particle production (Fig. 2G). These gain- and loss-of-function experiments indicate that autophagic membrane generation assists infectious EBV particle production.

### 2.3 Macroautophagy Inhibition Leads to an Accumulation of EBV DNA in the Cytosol

**Fig. 3:**
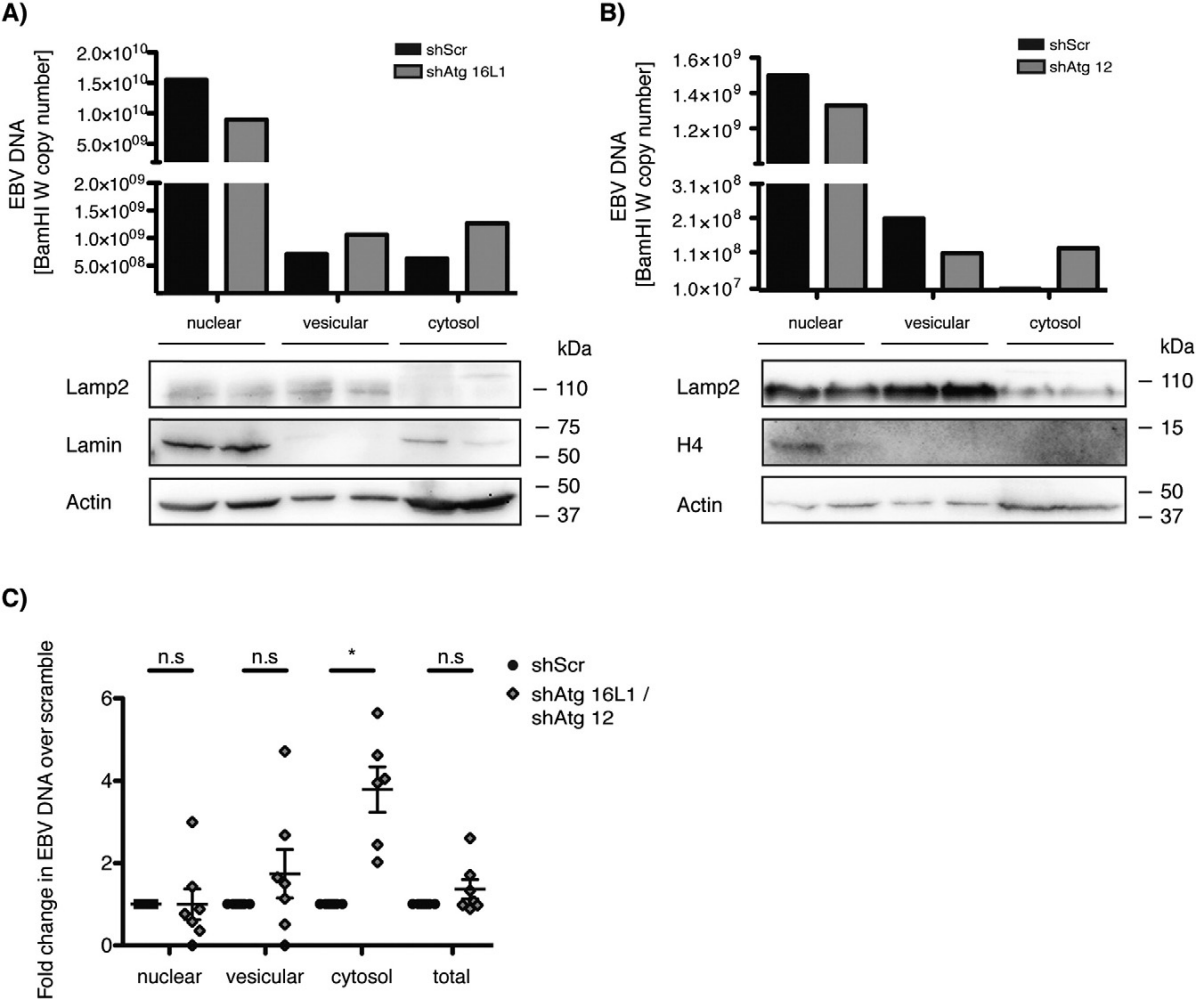
Macroautophagy down-regulation traps viral DNA in the cytosol. (A) and (B) Subcellular fractionation was performed on lytic 293/EBV-wt cells 3 days post-transfection, previously transduced with Atg12 specific, shAtg16 specific or scramble shRNA. Fractions were obtained by differential sequential centrifugation and EBV episome copies were determined by RT-qPCR. Viral copy number by BamH1 W fragment specific PCR is shown for the individual fractions on top, while distribution of vesicular (Lamp2), nuclear (Lamin or Histone 4 (H4)) and cytosolic (Actin) marker proteins as assessed by Western blot analysis is shown on the bottom. Representative data for six independent experiments is shown. (C) Data of (A) and (B) is expressed as ratio of viral loads of Atg16 or Atg12 specific shRNA to control (shScr) treated fractions. Scramble is set to 1. Statistics are performed on mean calculated copy numbers from CT values of triplicates from six independent experiments. Data are expressed as mean ± SD, paired *t* test; *, P < 0.05.

In order to understand at which step autophagic membranes assist lytic EBV replication, we investigated the intracellular distribution of EBV particles by their DNA in 293/EBV-wt cells. Macroautophagy inhibition did not cause substantial differences in the total intracellular EBV DNA as analyzed by real-time PCR (Fig. 3C, total). Hence, after cellular fractionation we analyzed the different subcellular compartments for their EBV DNA content. As expected, EBV copy numbers were highest in the nuclear fraction, the site of viral DNA replication. Nevertheless, we did not detect significant differences in viral genome content in the nuclear and vesicular fractions of 293/EBV-wt cells transfected with control versus macroautophagy silencing constructs (Fig. 3A to C). In contrast, we could see a significant increase in EBV DNA content in the macroautophagy deficient cytosolic fraction, when comparing to the control transduced cells (Fig. 3A to C), indicating that EBV particles accumulate in the cytosol upon macroautophagy inhibition. These studies suggested that autophagic membranes might contribute to cytosolic maturation of EBV.

### 2.4 LC3-II is Present in Purified EBV Viral Particles

**Fig. 4:**
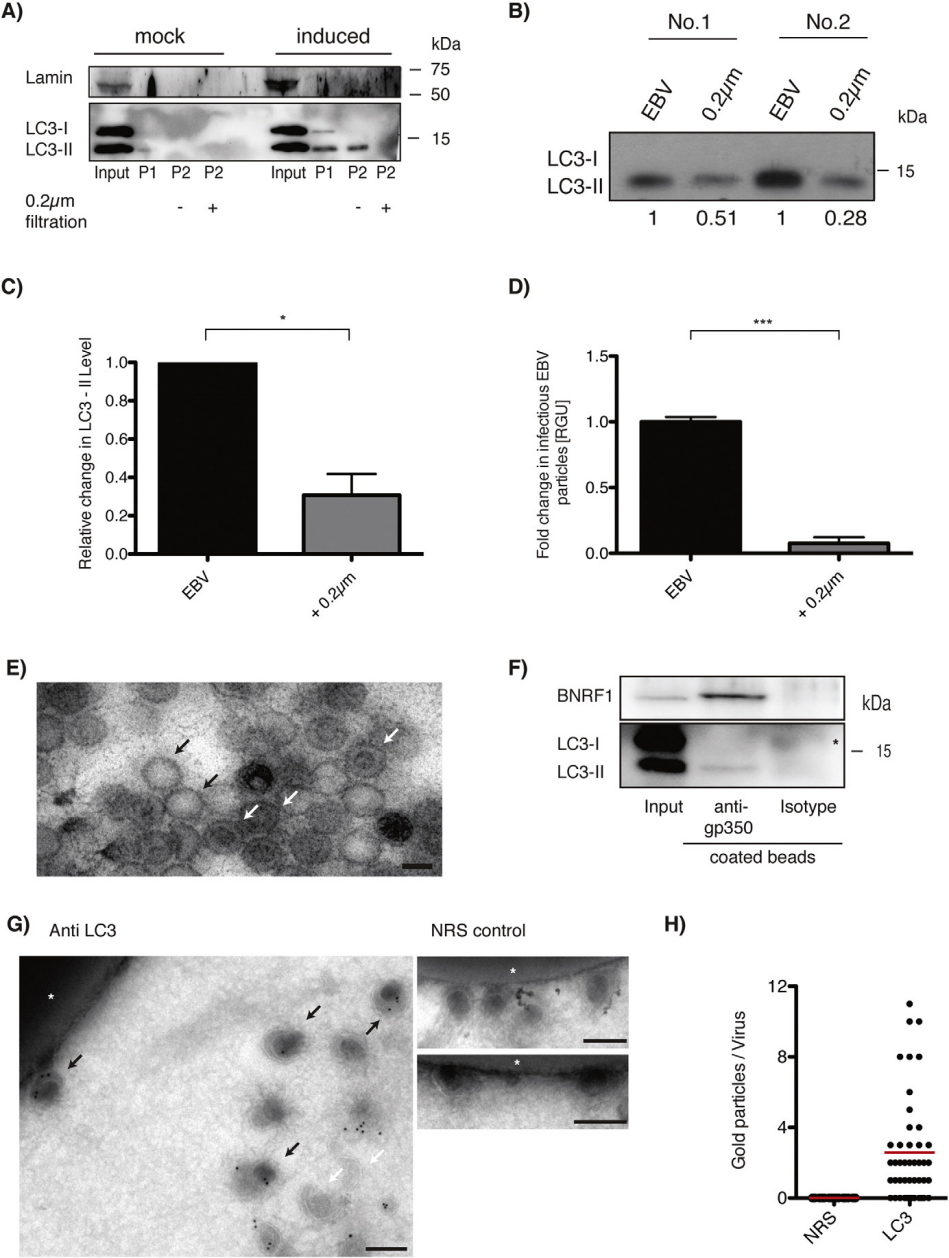
LC3-II is found in viral particles. (A) Supernatants obtained from mock and induced 293/EBV-wt cells were harvested after 3 days and subjected to purification by sequential centrifugation steps. Total cell lysate (input), cellular debris (P1) and virus containing pellets (P2) were subjected to Western blot analysis for Atg8/LC3 and Lamin A/C. Moreover, P2 was resuspended and one part was further filtered through 0.2 μm pore filters to remove EBV (+) and the other part was left unfiltered (−). Western blot results are representative of a total of six performed experiments. (B) Two independently performed experiments with LC3 protein evaluation of P2 pellets by Western blot, with and without 0.2 μm filtration. (C) Densitometry quantification of LC3-II protein level of six experiments (including A and B). Statistics are performed on relative units (RU), paired *t* test *, P < 0.05. (D) Raji titration on filtered (+ 0.2 μm) and unfiltered (EBV) P2 fractions. Data is represented in fold change to EBV fraction for three performed experiments with median ± SD, paired *t* test on RGU/ml, ***, P < 0.001. (E) Transmission electron microscope picture of viral particles precipitated with anti-gp350 antibody coupled beads. Mature virus particle (white arrow) and capsid depleted virus like particles (black arrow). Scale bar 100 nm. (F) Western blot analysis for Atg8/LC3 and BamH1 N fragment rightward reading frame 1 (BNRF1) viral tegument protein of total cell lysate (input), anti-gp350 bead precipitated virus (anti-gp350) and precipitates that were obtained with isotype coupled beads as a control (isotype). Blot represents 3 individually performed experiments. (G) Immunoelectron microscopic picture of anti-gp350 bead precipitated virus, developed with gold particle coupled secondary antibodies recognizing Atg8/LC3 specific antiserum (left, anti LC3). Black arrows indicate some of the precipitated viruses, white asterisk one antibody coupled bead and white arrows non-viral membrane aggregates. Two representative images are shown for the rabbit control serum (right, NRS control). Scale bar = 200 nm. (H) Quantification of gold particles per virus of (G) in LC3 stained versus NRS control. Data is represented as median by horizontal red line.

Indeed we could find in transmission electron micrographs cytosolic viral capsids in lytically EBV replicating 293/EBV-wt cells that were surrounded by two membranes, reminiscent of autophagosomes (Supplementary Fig. 7). If these autophagic membranes would contribute to cytosolic viral particle maturation by providing the second and the final envelope of EBV, then one would have to postulate that Atg8/LC3, which remains covalently coupled to the inner autophagosomal membrane, could be found in purified viral particles. Therefore, we enriched recombinant EBV from supernatants of induced 293/EBV-wt cells by sequential centrifugation and blotted virus pellets against LC3 (Fig. 4A, P2, and B). As controls, we used virus-depleted, filtered supernatants and non-induced 293/EBV-wt supernatants (Fig. 4A, P2 + and mock P2, and B). Remarkably, we could detect high levels of the lipidated LC3-II, but not free LC3-I in the virus pellet, whereas no LC3 was present in the non-induced supernatants (Fig. 4A to D).

In order to exclude the co-purification of LC3 containing vesicles of similar density and size, we visualized LC3 on purified virus particles by means of immunoelectron microscopy (Fig. 4G). For this purpose, we precipitated EBV particles with anti-gp350 coated beads, which enriched for morphologically identifiable virus particles (Fig. 4E) and lipidated LC3 (Fig. 4F). Morphological analysis revealed that VLPs (Fig. 4E, black arrows) and mature virions (Fig. 4E, white arrows) are present in the precipitation of EBV. Immunogold-labeling of LC3 revealed the presence of this label of autophagic membranes on virus particles (Fig. 4G, black arrows), while no background could be observed on the beads (asterisk) or membrane aggregates (Fig. 4G, white arrows). Moreover, control rabbit antiserum did not label viral particles (Fig. 4G, NRS control), and the LC3 specific antiserum significantly labeled enriched viral particles compared to rabbit control serum (Fig. 4H). Thus, lipidated LC3-II seems to get incorporated into EBV viruses during their maturation in the cytosol and these data strongly indicate that autophagic membranes contribute to the final envelope of this ubiquitous human tumor virus.

## 3 Discussion

Our study demonstrates the incorporation of membranes that have been modified by the macroautophagy machinery into virus particles. This membrane reorganization seems to assist the envelope acquisition of EBV in the cytosol, as macroautophagy inhibition decreases infectious particle production and accumulates EBV DNA in the cytosol. Therefore, autophagic membrane stabilization, which we observed during the induction of lytic EBV replication and could be further elevated via rapamycin, augments EBV production.

While direct recruitment of autophagic membranes into virus particles has not been reported before, a number of viruses seem to target the catabolic macroautophagy pathway. Indeed, only Sindbis virus has been shown to be directly restricted by macroautophagy mediated degradation (Orvedahl et al., 2011). Double-membrane vesicle accumulation after poliovirus infection was already shown in 1965 (Dales et al., 1965). This and other RNA viruses seem to replicate on membranes, which they induce via the macroautophagy machinery (Dreux et al., 2009, Jackson et al., 2005, Judith et al., 2013). Some also use the induced vesicular compartments to leave their host cells, as has been proposed for HIV replication in myeloid cells (Kyei et al., 2009). Such accumulation of autophagic membranes can result from pathogen-induced inhibition of autophagosome fusion with lysosomes. Such an accumulation was shown for influenza A virus infection (Gannage et al., 2009), and some of the enriched Atg8/LC3 coupled membranes might be redirected to the plasma membrane for filamentous budding of stable virus particles (Beale et al., 2014). However, the utilization of autophagic membranes has not been reported so far for DNA viruses. Indeed, herpes viruses rather seem to encode Bcl-2 homologues that inhibit autophagosome formation via their binding to Atg6/Beclin-1 (E et al., 2009, Ku et al., 2008, Orvedahl et al., 2007, Pattingre et al., 2005). EBV also encodes two Bcl-2 homologues, BamH1 H fragment rightward reading frame 1 (BHRF1) and BamH1 A fragment leftward reading frame 1 (BALF1), but their influence on macroautophagy has not been reported (Altmann and Hammerschmidt, 2005). Nevertheless, they could be responsible for the initial macroautophagy inhibition that we observed early after lytic EBV replication induction in AKBM cells (Supplementary Fig. 4A and B). In addition, viral FLICE-like inhibitor proteins (vFLIPs) of herpes viruses seem to inhibit binding of Atg3 to Atg8/LC3, thereby inhibiting the E2-like step of Atg8/LC3's ubiquitin-like conjugation to membranes (Lee et al., 2009). The human γ-herpes virus Kaposi Sarcoma associated herpes virus (KSHV) encodes both a macroautophagy inhibiting Bcl-2 and a Atg3 binding FLIP homologue (Lee et al., 2009, Pattingre et al., 2005), but seems to increase macroautophagy after Rta mediated reactivation of its lytic replication (Wen et al., 2010). In contrast to the capacity of most herpes viruses to inhibit macroautophagy, the other human γ-herpes virus EBV seems to utilize the molecular machinery of macroautophagy. Along these lines, the latent membrane protein 1 (LMP1) of EBV seems to stimulate macroautophagy (Lee and Sugden, 2008). With this up-regulation LMP1 adjusts its expression levels via autophagic LMP1 degradation. In the absence of this degradation regulation, EBV transformed B cells undergo apoptosis due to high LMP1 expression. Furthermore, it was recently shown that the immediate early lytic EBV antigen BamH1 R segment leftward reading frame (BRLF1) up-regulates macroautophagy (Hung et al., 2014). Moreover, lytic EBV replication inhibits autophagosome degradation to benefit its own virus particle production (Granato et al., 2014). In addition, we now demonstrate that EBV stabilizes autophagic membranes during lytic infection and uses these membranes for its envelope during cytosolic maturation. We could demonstrate that Atg8/LC3-II is contained in supernatant fractions of lytically EBV replicating cells similar in density and size to viral particles. Moreover, the precipitation of the EBV envelope protein gp350 with antibody-coupled beads enriched the main tegument protein BNRF1 and Atg8/LC3-II compared to a control antibody. Finally immunoelectron microscopy revealed the presence of Atg8/LC3 in herpes virus-like particles in the supernatant of lytically EBV replicating cells. These five lines of evidence suggest that EBV incorporates autophagic membranes into its viral particles. Therefore, EBV seems to be a DNA virus that uses the macroautophagy machinery for its replication.

However, leaving macroautophagy intact might render EBV vulnerable to detection by adaptive T cell responses after antigen processing via macroautophagy (Münz, 2009). Indeed, the nuclear antigen 1 of EBV (EBNA1) has been found to gain access to MHC class II presentation intracellularly via macroautophagy (Leung et al., 2010, Paludan et al., 2005). The resulting CD4^+^ T cell recognition leads to the elimination of EBV transformed B cells in vitro (Long et al., 2005, Nikiforow et al., 2003, Paludan et al., 2002). The underlying antigen processing pathway seems to involve cytosolic engulfment of EBNA1 and fusion of autophagosomes with MHC class II loading compartments (MIICs), which are primarily late endosomes equipped with the molecular machinery for peptide generation and binding to MHC class II molecules (Schmid et al., 2007). Consistent with EBV particles localizing to autophagic membranes and then gaining access to MHC class II loading is the finding that late lytic EBV antigens are frequently recognized by CD4^+^ T cells, while CD8^+^ T cell responses mainly focus on immediate early and early lytic EBV antigens (Hislop et al., 2007). While antigen release and up-take by neighboring latent EBV infected cells has been identified as one antigen processing pathway to stimulate these late EBV antigen specific CD4^+^ T cells (Adhikary et al., 2006), lytically EBV replicating cells might be recognized directly via intracellular antigen processing onto MHC class II molecules. Thus, usurping macroautophagy for cytosolic enveloping might be a very efficient strategy of EBV to acquire a membrane coat in the cytosol, but might also in part result in the presentation of its late lytic antigens onto MHC class II molecules.

In summary, our study suggests that EBV uses Atg8/LC3-coupled membranes to assemble its second envelope in the cytosol. This process seems to be required for efficient production and release of infectious EBV particles. It represents an example of a human DNA virus directly using the macroautophagy machinery to build its infectious particles and to incorporate Atg8/LC3 coupled membranes into them.

## 4 Material and Methods

### 4.1 Cell Culture

Akata cell subclones; EBV-positive AKBM and EBV-negative AK31 (Ressing et al., 2005) were cultured in R10 medium (RPMI + 10% heat-inactivated FCS (PAA) + 50 U/ml penicillin/streptomycin); AKBM cells were additionally grown in selection medium containing hygromycin (300 μg/ml). Ligation of cell surface immunoglobulin (IgG) was conducted by adding 25 μg/ml of goat-anti human anti-F(ab′)_2_ IgG for 2 h, followed by the addition of fresh R10 in a total of 4× the initial volume and further incubation for 20 h at 37 °C, 5% CO_2_. Cells considered for immunoblot analysis were either enriched populations via MACS sorting or at least 70% GFP-positive. Sorting for lytic cells was conducted by a mouse anti-ratCD2 specific antibody (OX34) and then positively selected with goat anti-mouse IgG2a/b microbeads and MS columns (Miltenyi). 293/EBV-wt cells were a generous gift from the Delecluse lab (DKFZ, Heidelberg, Germany). Briefly, these cells contain the recombinant EBV strain B95-8 DNA as a bacmid under the selection of hygromycin. Cells were cultured in D10 (DMEM + 10% heat-inactivated FCS + 20 μg/ml gentamycin + 20 μg/ml hygromycin). EBV production was triggered by transfection of 293/EBV-wt cells with BZLF1 (p509) and BALF4 (p2670) plasmids. In brief, small-scale experiments were conducted with 0.75 × 10^6^ cells in a 6 cluster-well plate with 0.5 μg of each plasmid and the metafectene (Biontex) mix was incubated on cells for 3 h and replaced with 2 ml of fresh D10. EBV viral supernatants were harvested 3 days post-transfection, if not stated otherwise.

### 4.2 Antibodies

For immunoblotting, anti-Atg8/LC3 (clone 5F10) and -Atg5 (clone 7C6) were purchased from Nanotools, Lamp2 (clone H4B4) from Southern Biotech, mouse anti β-actin (clone AC-15) from Abcam and Lamin A/C (clone H-110) from Santa Cruz Biotechnologies and antibody anti-histone H4 (clone 62-141-13) was obtained from Millipore. For immunofluorescence stainings, rabbit anti-Atg8/LC3 was received from Medical Biological Laboratories, mouse anti-gp350 (clone 72A1, HB) from hybridoma cells from ATCC, and mouse anti-BZLF1 (clone BZ-1) from Santa Cruz Biotechnologies. Secondary antibodies conjugated with Alexa Fluor 555 or Alexa Fluor 647 were purchased from Invitrogen. Control rabbit polyclonal antiserum used for immunoprecipitation was obtained from Novus Biological.

### 4.3 Lysate Preparation and Immunoblotting

In order to obtain protein extracts, cells were collected at the indicated times, washed twice in PBS, resuspended in ice-cold lysis buffer (1% NP-40 with complete protease inhibitor cocktail from Roche) and incubated on ice for 10 min. The resulting cell lysates were centrifuged at 20,000 *g* for 10 min at 4 °C. For all samples, equal amounts of total protein extracts were boiled for 5 min in the presence of SDS-PAGE loading buffer NuPage (Life Technologies) with 1% β-mercaptoethanol. Protein extracts were resolved in 12.5% SDS-PAGE gels and transferred onto PVDF membranes (GE healthcare). For the detection of specific protein bands, primary antibodies, HRP-conjugated secondary antibodies and the ECL Plus detection systems were used (Thermo Scientific). Quantification of protein levels by densitometry was performed on a Vilber Lourmat Fusion FX imaging system or on digitalized films using the ImageJ software.

### 4.4 Quantitative PCR

Cellular DNA was extracted using QIAamp DNeasy blood and tissue kit and viral DNA from culture supernatant was obtained with the QIAamp minELUTE viral DNA kit (Quiagen). EBV DNA was quantified by Taqman (Applied Biosystems) Real-time PCR with BamHI-W fragment primers (Fw 5′-GGACCACTGCCCCTGGTATAA-3′; Rev 5′-TTTGTGTGGACTCCTGGGG-3′; Probe 5′-(6FAM)-TCCTGCAGCTATTTCT GGTCGCATCA-(TAMRA)-3′) and was performed on a CFX384 Touch Real-Time PCR Detection System (Bio-Rad). All samples were tested in triplicates, and mean results were determined.

### 4.5 Immunofluorescence

AKBM cells were centrifuged (500 *g*, 3 min) on poly-l-lysine coated glass slides (Menzel-Gläser; 1.5 mm) and 293/EBV-wt cells were cultured on poly-d-lysine coated round glass coverslips in a 12 well plate. At the indicated time points, cells were fixed in 3% PFA for 15 min at 4 °C. All subsequent steps were performed at room temperature and all washes done with PBS supplemented with 1% fish skin gelatin and 0.02% saponin (Sigma). Cells were permeabilized with 0.1% Triton X-100 for 5 min and then incubated with signal enhancer (Image-iT FX, Invitrogen), followed by staining with the indicated antibodies and suitable secondary antibodies. Slides were counterstained with DAPI and mounted with ProLong gold antifade reagent (Invitrogen). Cells were visualized through a 63 ×, 1.4 NA oil immersion lens with an inverted confocal laser-scanning microscope (SP5; Leica). Counted cells are represented as dot plots with median values displayed as a horizontal line.

### 4.6 ShRNA Silencing Through Lentiviral Transduction

The shRNA expression plasmids pLKO.1-puromycin shRNA scramble (5-TCCTAAGGTTAAGTCGCCCTCG-3) and atg16 (5-CAGGAAGCCAATCGGCT TAAT−3) were gifts from the J. Tschopp lab (University of Lausanne, Lausanne, Switzerland). The shRNA atg12 (5-CCAAGGACTCATTGACTTCAT-3) was a gift from the F.V. Chisari lab (Scripps Research Institute, La Jolla, USA). Production of lentiviral particles was performed as previously described in Schmid et al. (2007). In brief, lentiviral vectors were co-transfected with the helper plasmids pCMV_R8.91 and pMD.G into 293T cells by calcium phosphate transfection. Cell culture supernatants containing the recombinant viral particles were harvested on day 2 post-transfection, filtered through a 0.22 μm pore filter, and frozen at − 80 °C. For silencing with shRNA recombinant constructs, 293/EBV-wt cells were infected with the corresponding lentiviruses at an MOI of 5 and incubated for a total of 3 days. Cells were kept on selection medium (puromycin 4 μg/ml) for 2 passages before being used in experiments.

### 4.7 Electron Microscopy

EBV particles purified with anti-gp350 coated microspheres were fixed for 16 h at room temperature (RT) with 3% formaldehyde followed by 2 h 2.5% glutaraldehyde in 0.1 M NaPO_4_ (pH 7.4) and, subsequently, with 1% OsO4 in 50 mM sodium cacodylate buffer, pH 7.3 dehydrated in an ethanol series and embedded into Epon (Catalys). Ultrathin sections of 50 nm were contrasted with uranyl acetate and lead citrate. For immunostaining EBV particles on anti-gp350 coated beads were fixed with 3% formaldehyde, 0.025% glutaraldehyde in 0.1 M NaPO_4_ (pH 7.4), and after three washing steps in PBS embedded in 12% gelatin in 0.1 M NaPO_4_ (pH 7.4). Small pieces were incubated for 16 h in 2.3 M sucrose in 0.01 M NaPO_4_ (pH 7.4) and frozen in liquid nitrogen. Cryosections were thawed and incubated after blocking with 0.5% BSA (Applichem), 0.15% gelatin (Sigma) in 0.1 M NaPO_4_ (pH 7.4; blocking buffer) using a rabbit anti LC3 antibody (MBL) followed by a goat anti rabbit Gold 12 nm, both in blocking buffer. After postfixation with 0.5% glutaraldehyde in H_2_O and three times washing in H_2_O sections were contrasted with 0.3% uranylacetate (Mallinkrodt), 1.8% methylcellulose in H_2_O at 4 °C. Sections were analyzed in a Tecnai Spirit transmission electron microscope (FEI) with an ORIUS CCD camera (Gatan).

### 4.8 EBV Purification

293/EBV-wt cells were transfected to produce EBV for 3 days, after which the supernatant was collected and filtered through a 0.45 μm filter. To obtain an enriched EBV pellet, supernatants were spun at 30,000 *g* for 2 h at 4 °C. The supernatant was carefully removed and the virus pellet was resuspended in 10 ml ice-cold PBS. This fraction was equally split into 2 tubes, one part was further filtered through a 0.2 μm filter, that retains EBV particles and the other left unfiltered. Both, 0.2 μm filtered and unfiltered supernatants, were further centrifuged at 30,000 *g* for 2 h at 4 °C and the resulting pellets were carefully resuspended in 100 μl ice-cold PBS. These fractions were examined by Raji titration (described below) for EBV content and their Atg8/LC3 protein levels were analyzed by Western blot.

### 4.9 Infectious Unit Determination (RGU)

Viral titers were determined from supernatants of 293/EBV-wt cells producing EBV throughout 3 days. In order to estimate infectious units in supernatants of EBV producing cells, the supernatant was filtered through 0.45 μm filters and inoculated on Raji or Ramos cells (4 × 10^4^) for 48 h as indicated. Titrations were always performed in duplicates. Cells were analyzed by flow cytometry using a BD FACS Canto Flow Cytometer and the FlowJo software. Dead cells were excluded through Live/Dead staining (Invitrogen) and EBV infected Raji cells were detected in the GFP-positive gate.

### 4.10 Inhibitors, Chemicals

Chloroquine and rapamycin were purchased from Sigma. Chloroquine was applied at a concentration of 50 μM, 6 h prior to the harvest of cells for Western blot. Rapamycin was applied 4 h prior to the induction of EBV production in 293/EBV-wt cells at the indicated concentrations.

### 4.11 Subcellular Fractionation

Cells were rinsed off culture dishes 3 days post-transfection with ice-cold PBS and washed twice. The resulting pellet was resuspended in 1 ml homogenization buffer (0.25 M sucrose, 1 mM EDTA, 20 mM HEPES, protease inhibitor cocktail, pH 7.4) and mechanically lysed by passing the extract 20 times through a 24 G needle. To obtain the various fractions, the lysate was consecutively spun at 1000 *g* for 10 min, 3000 *g* for 10 min and 17,000 *g* for 15 min to obtain the nuclear, mitochondrial (discarded) and vesicular fraction, respectively. The remaining supernatant contained the cytosolic portion. The resulting pellets were resuspended in PBS and equally divided into 2 tubes and re-pelleted. One part was subjected to Western blot analysis and the other part was resuspended in 200 μl of PBS and subjected to DNA extraction by QIAamp DNA extraction kit.

### 4.12 ELISA

For IL-10 detection, supernatants of AKBM and AK31 cells were harvested after 20 h. These were then added undiluted onto previously coated 96-well ELISA plates (Nunc-Immuno MaxiSorp; Thermo Fisher Scientific). Cytokines were then detected with the biotinylated specific antibodies and streptavidin–HRP (human IL-10 kit, Mabtech, Sweden), using the peroxidase substrate tetramethylbenzidine (Sigma). Recombinant human IL-10 (25–1000 pg/ml) was used as standard.

### 4.13 Cell Viability Assay

Cells were harvested at indicated time points, washed in PBS, resuspended in 1× binding buffer, and stained with 1 μl Annexin V (Biolegend) per 50 μl staining volume. 1 μl 7-AAD (Biolegend) per 50 μl staining volume was added to evaluate dead cells. Samples were incubated for 30 min at 4 °C and analyzed by flow cytometry on an LSR Fortessa flow cytometer (BD Biosciences). Annexin V was detected in the APC channel and 7-AAD in the PerCP channel.

### 4.14 Statistical Analysis

Where indicated, paired *t* tests were performed using the Prism software (version 5.0a; GraphPad Software).

## Conflict of Interest

The authors have no conflicting financial interests.

## Author Contributions

HN and BG performed and analyzed the experiments. BG performed the electron microscopy. SR, UZ and CM coordinated the project and planned the experiments. KT and JD contributed to the interpretation of the obtained experimental data. HN and CM wrote the manuscript with input from the other authors.

## Appendix A Supplementary Data

Supplementary Fig. 1. Induction efficacy of IgG cross-linked AKBM cells. Human AKBM cells were incubated for 2 h with anti-human IgG F(ab)_2_ fragments and cultured in fresh medium for 20 h. Lytic induction efficacy was assessed by flow cytometry (FACS). GFP presence indicated the AKBM cells undergoing lytic reactivation (+ IgG, upper panel). FACS plots represent at least 12 performed experiments. Supplementary Fig. 2. Supernatants of stimulated (+ IgG) or non-stimulated (− IgG) AKBM and AK31 cells (incubated for 2 h with anti-human IgG F(ab)_2_ fragments and cultured in fresh medium for 20 h) were subjected to IL-10 specific ELISA assays. Data is represented as means ± SD (n = 4). Supplementary Fig. 3. Immunofluorescence analysis of Atg8/LC3 of latent and lytic AKBM cells. (A) RatCD2–GFP on the cell surface (green), Atg8/LC3 (white) and DAPI (blue) were assessed. Two representative cells are shown for each condition. Scale bar 2 μm. (B) Quantification of LC3 positive puncta in stimulated (+ IgG, ratCD2–GFP^+^) versus non-stimulated (− IgG, ratCD2–GFP^−^) AKBM cells. Supplementary Fig. 4. Time course of macroautophagy up-regulation during lytic EBV replication. (A) AKBM cells were induced to replicate EBV by BCR cross-linking. Aliquots were taken at the indicated time points after induction and subjected to actin, BZLF1 and Atg8/LC3 specific Western blotting. The LC3-II signal was normalized to actin and its ratio to time point 0 is shown below the panels. (B) Time course as in (A) for IgG cross-linked (+) and untreated (−) AKBM cells. Lytic EBV replicating cells were MACS sorted as ratCD2 positive and lysates were assessed for their actin and Atg8/LC3 content by Western blotting. Asterisk indicates light chain of mouse anti-ratCD2 antibody used in the MACS sorting processing. (C) 293/EBV-wt cells were transfected for 3 h and cells were harvested at indicated time points post-transfection and their lysates were subjected to actin, BZLF1 and Atg8/LC3 specific Western blotting. Ratio of LC3-II normalized to actin compared to time point 0 is depicted below the blot. Blot represents 2 individually performed experiments. Supplementary Fig. 5. Immunofluorescence analysis of Atg8/LC3 dots in 293/EBV-wt cells 24 h and 48 h post-lytic induction. 293/EBV-wt cells were transfected and fixed after 24 h and 48 h post-induction and analyzed by immunofluorescence microscopy. (A) Atg8/LC3 positive dots were counted in mock and BZLF1 positive cells after 24 h and 48 h. EBV production efficacy was assessed by BZLF1 positivity, which was at least 60%. Data is represented as median by horizontal red line. (B) Representative Images for (A). Scale bar 10 μm. Supplementary Fig. 6. Viability of 293/EBV-wt cells after transduction with scramble and Atg specific shRNA encoding lentiviruses. (A) Annexin V/7-AAD staining. Induced 293/EBV-wt cells were stained 3 days post-transfection with Annexin V for apoptotic marker phosphatidylserine on the cell surface by FACS, DNA accessibility was counterstained by 7-AAD. Bar graph represents two independently performed FACS staining analysis. (B) 293/EBV-wt cells were counted after removal of supernatant containing virus. Data represents cell counts of 5 experiments for the atg12 silencing and 6 assays for the atg16 silencing. Bars represent means ± SD. Supplementary Fig. 7. Maturation of EBV particles. I: Free capsids in the nucleus; II: Possibly first enveloping of viral capsids at the nuclear membrane, III: Viral capsid surrounded by two membranes (possibly autophagosome) in the cytosol; IV: Enveloped virus in the supernatant. Scale bar: 1 μm.
