# Supplementary material for: Macroautophagy Proteins Assist Epstein Barr Virus Production and Get Incorporated Into the Virus Particles
Source: eBioMedicine. 2014 Nov 8;1(2-3):116–25. doi: 10.1016/j.ebiom.2014.11.007 (PMC4457436; doi:10.1016/j.ebiom.2014.11.007)

Supplementary Figure 1

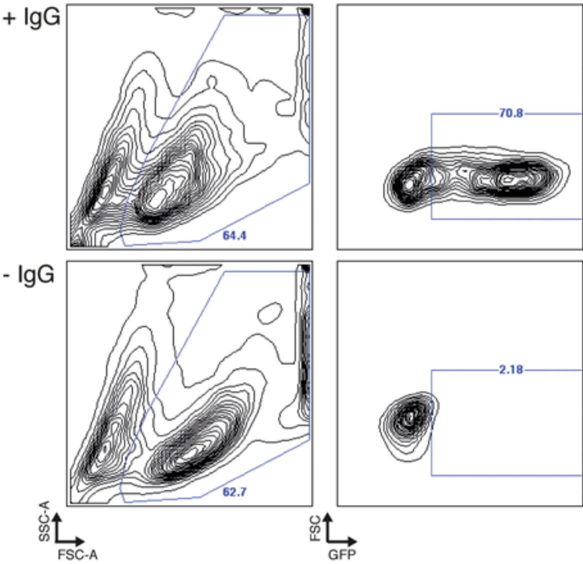

Supplementary Figure 2

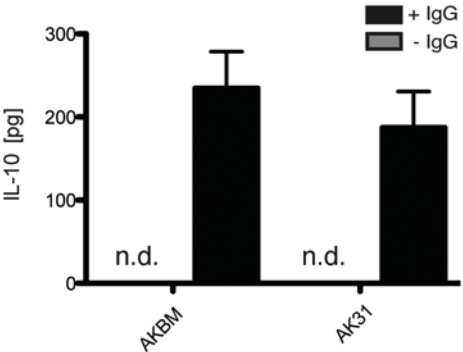

Supplementary Figure 3

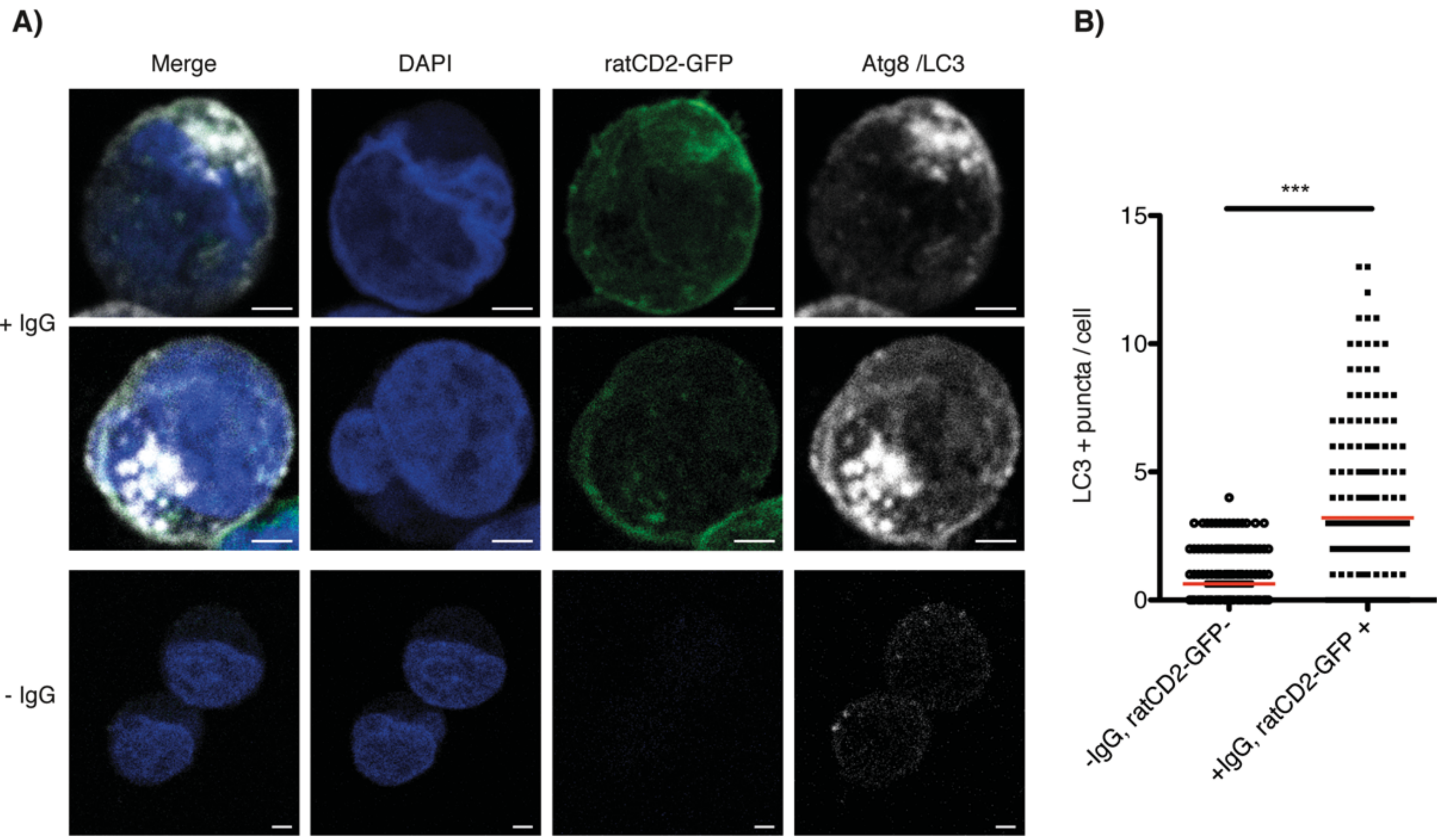

Supplementary Figure 4

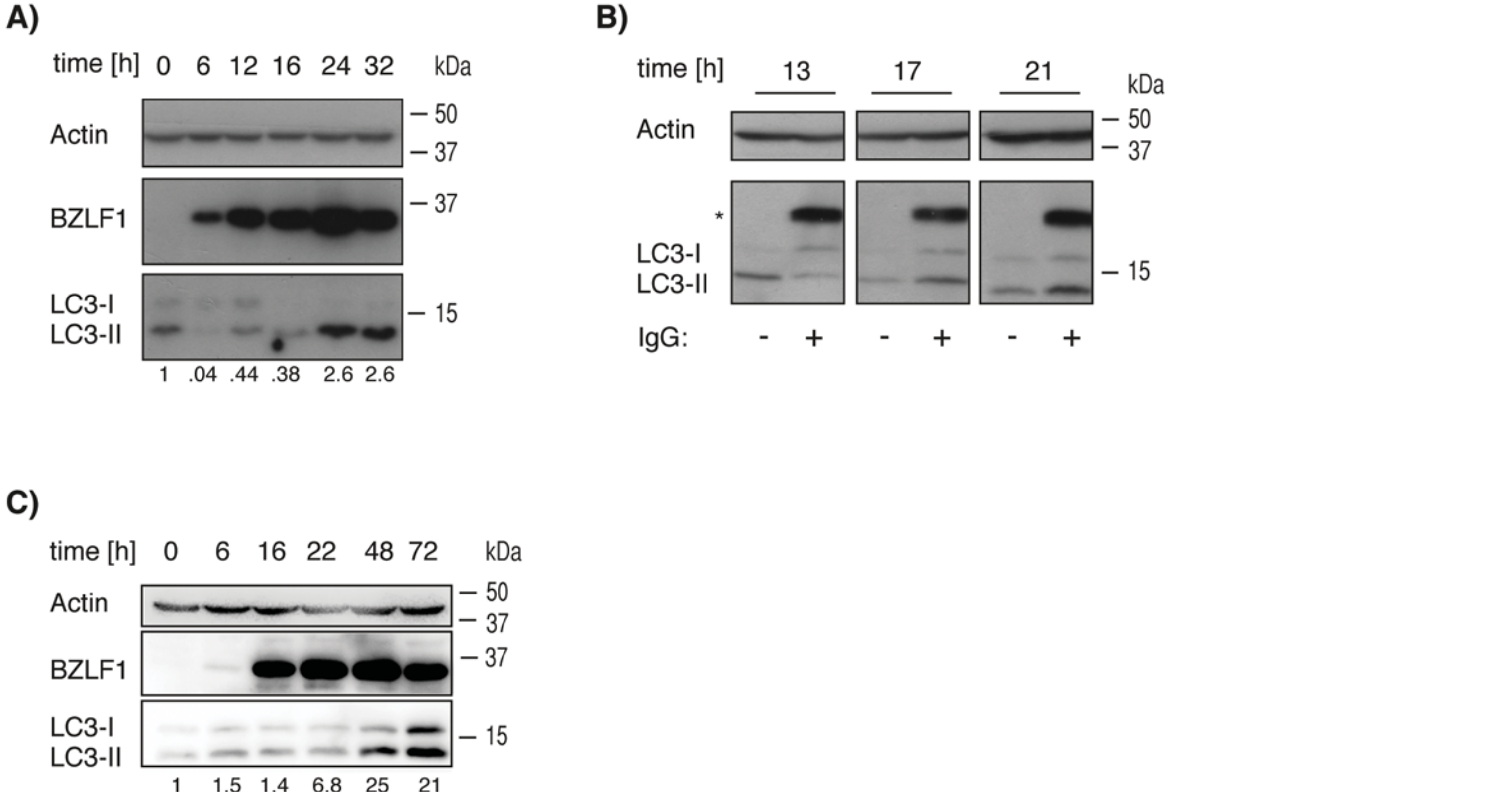

Supplementary Figure 5

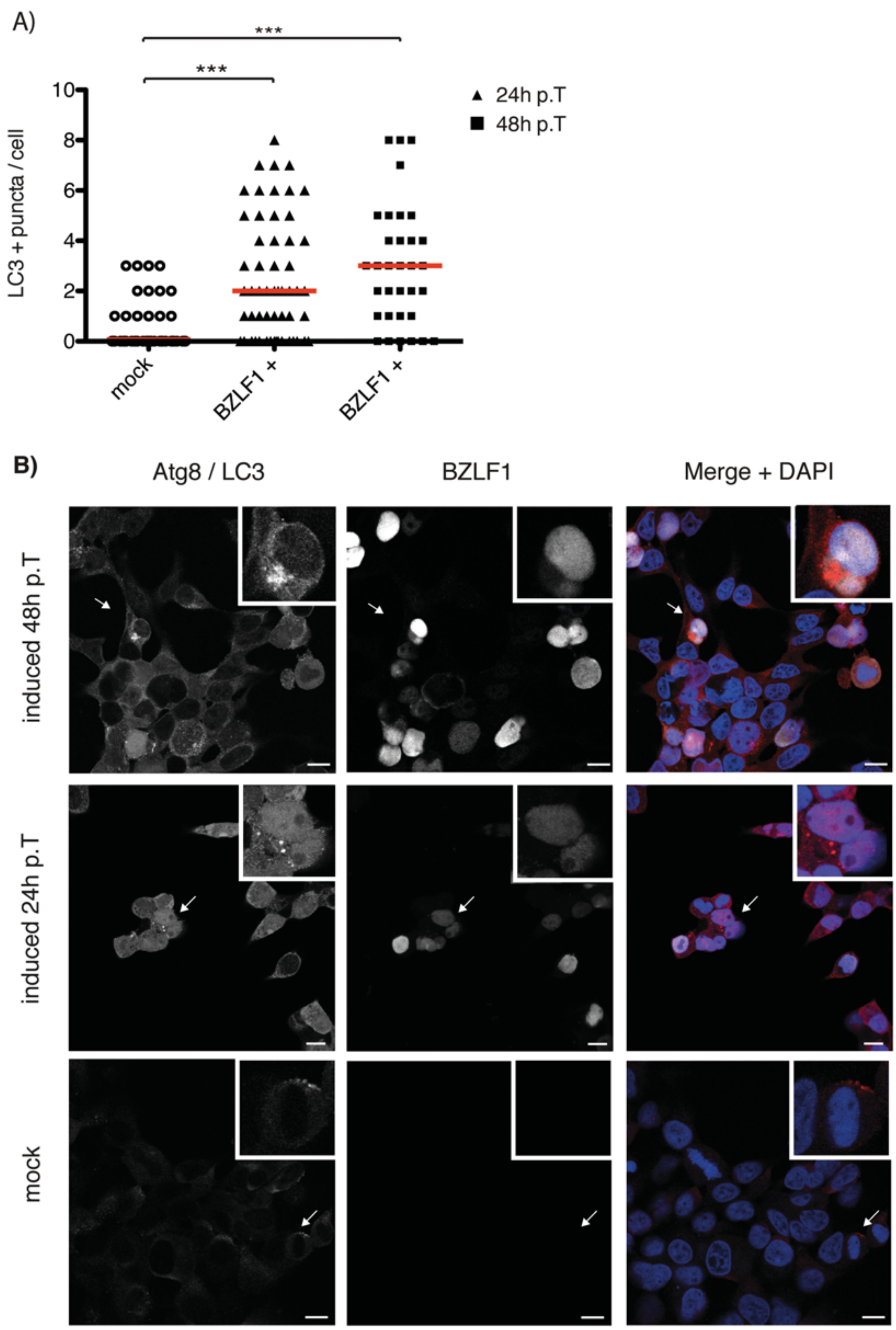

Supplementary Figure 6

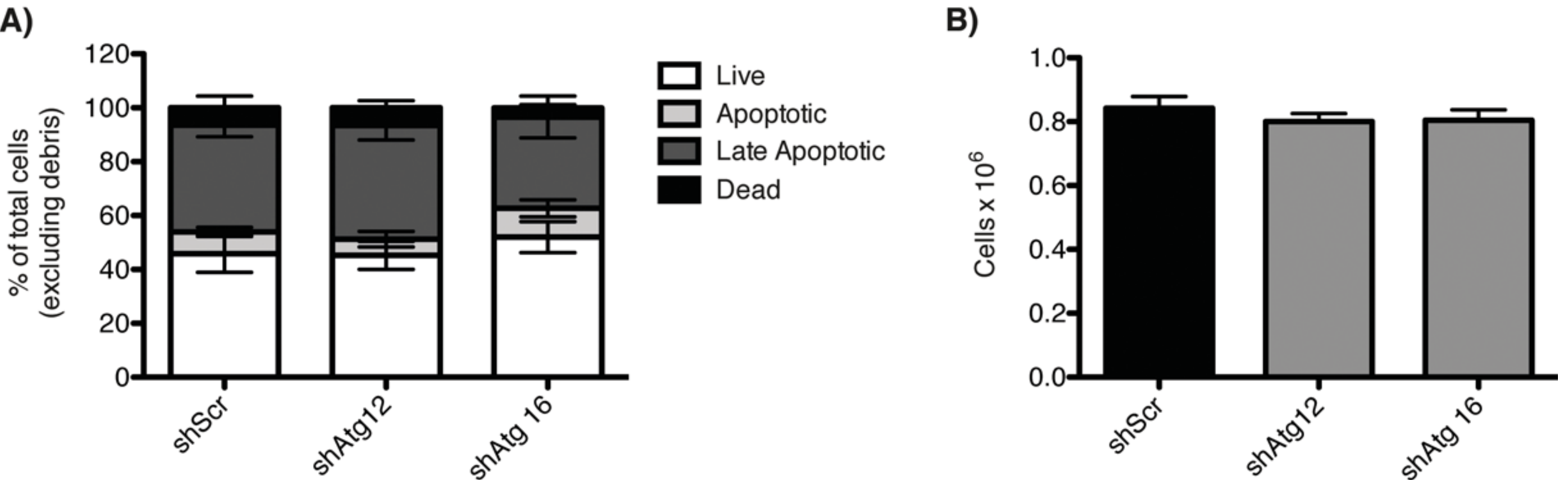

Supplementary Figure 7

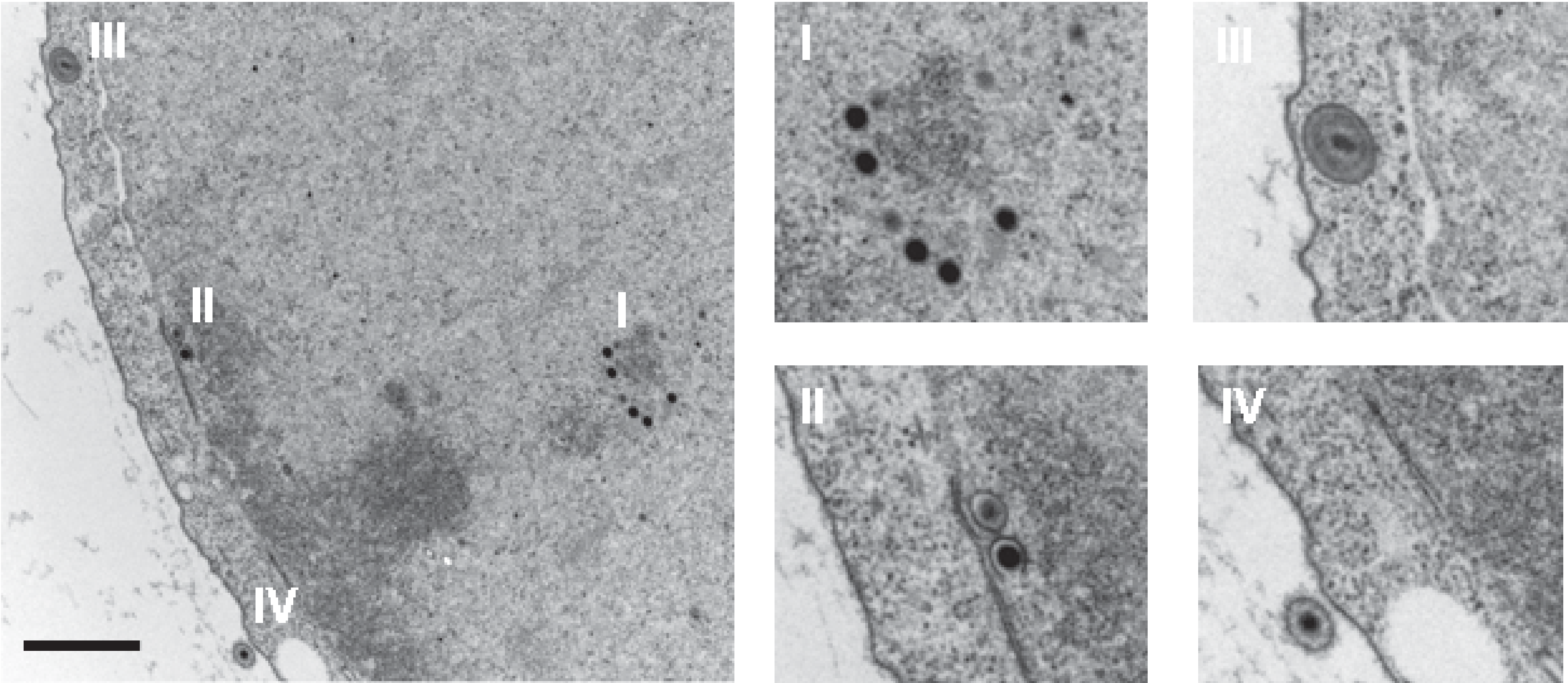

Supplement: Supplementary file 1 — Supplementary Fig. 1. Induction efficacy of IgG cross-linked AKBM cells. Human AKBM cells were incubated for 2 h with anti-human IgG F(ab)2 fragments and cultured in fresh medium for 20 h. Lytic induction efficacy was assessed by flow cytometry (FACS). GFP presence indicated the AKBM cells undergoing lytic reactivation (+ IgG, upper panel). FACS plots represent at least 12 performed experiments. Supplementary Fig. 2. Supernatants of stimulated (+ IgG) or non-stimulated (− IgG) AKBM and AK31 cells (incubated for 2 h with anti-human IgG F(ab)2 fragments and cultured in fresh medium for 20 h) were subjected to IL-10 specific ELISA assays. Data is represented as means ± SD (n = 4). Supplementary Fig. 3. Immunofluorescence analysis of Atg8/LC3 of latent and lytic AKBM cells. (A) RatCD2–GFP on the cell surface (green), Atg8/LC3 (white) and DAPI (blue) were assessed. Two representative cells are shown for each condition. Scale bar 2 μm. (B) Quantification of LC3 positive puncta in stimulated (+ IgG, ratCD2–GFP+) versus non-stimulated (− IgG, ratCD2–GFP−) AKBM cells. Supplementary Fig. 4. Time course of macroautophagy up-regulation during lytic EBV replication. (A) AKBM cells were induced to replicate EBV by BCR cross-linking. Aliquots were taken at the indicated time points after induction and subjected to actin, BZLF1 and Atg8/LC3 specific Western blotting. The LC3-II signal was normalized to actin and its ratio to time point 0 is shown below the panels. (B) Time course as in (A) for IgG cross-linked (+) and untreated (−) AKBM cells. Lytic EBV replicating cells were MACS sorted as ratCD2 positive and lysates were assessed for their actin and Atg8/LC3 content by Western blotting. Asterisk indicates light chain of mouse anti-ratCD2 antibody used in the MACS sorting processing. (C) 293/EBV-wt cells were transfected for 3 h and cells were harvested at indicated time points post-transfection and their lysates were subjected to actin, BZLF1 and Atg8/LC3 specific Western blottin [file mmc1.pdf]
